# Temperature and the progression of developmental milestones in embryogenesis of a marine ectotherm

**DOI:** 10.1098/rsob.250062

**Published:** 2025-09-24

**Authors:** Emily K. Belcher, Travis K. Johnson, Christen Mirth, Keyne Monro

**Affiliations:** ^1^ School of Biological Sciences, Monash University, Melbourne, Victoria, Australia; ^2^ La Trobe University, Melbourne, Victoria, Australia

**Keywords:** developmental progression, developmental robustness, embryonic stages, polychaete worms, thermal responses

## Abstract

Embryos are among the most temperature-sensitive life stages. To survive and produce juvenile stages, embryos must be robust to changes in temperature that also change development time profoundly. Yet, how robustness is achieved during embryogenesis, and which developmental events are most prone to perturbation by temperature, is only known for a handful of species. Such insights are especially lacking for marine ectotherms, which often develop in direct contact with the external environment. We address these gaps using the tubeworm, *Galeolaria caespitosa*, a typical marine ectotherm with external development. We fluorescently labelled F-actin and nuclear DNA in embryos sampled hourly throughout embryogenesis at the minimum temperature of the coldest month (11°C), annual mean temperature (17°C) and maximum temperature of the warmest month (22°C) in nature. Based on confocal imaging, we identified key developmental stages (milestones) in embryogenesis and compared their progression across temperatures. We found that developmental progression is similar across temperatures when normalized to development time, but earlier milestones are less robust to warming than later ones. Our results suggest that embryos achieve robustness by tightly coordinating the relative timing of embryonic events, offering clues to how embryos may withstand contemporary climate change in marine systems.

**Figure:**
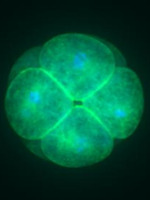

## 1. Introduction

Temperature sets the pace of life, from rates of energy flux as organisms develop, mature and reproduce, to the timing of these life events [1,2]. As rapid climate change exposes organisms to temperatures unseen in recent history [3], the formative events of embryogenesis may be prone to perturbation. This is a particular risk for ectotherms, whose embryos face the challenge of developing at ambient environmental temperatures without the thermoregulatory mechanisms (e.g. behaviour and cardiorespiratory systems) that combat thermal stress later in life [4,5]. The challenge is even more formidable for marine ectotherms, which live closer to their upper thermal limits and have less capacity to escape thermal stress than terrestrial ectotherms do [6,7]. Moreover, many marine ectotherms are external fertilizers that develop in direct contact with the external environment [8], increasing the vulnerability to thermal stress at critical life stages such as embryogenesis [5,9]. Because abnormal development lowers survival and reproduction, species cannot sustain healthy populations across their ranges unless embryogenesis is robust to changes in temperature [10]. Consequently, there is a need to better understand how robustness is achieved as embryos develop at different temperatures, especially in marine realms.

Development is said to be robust if morphological phenotypes at developmental endpoints (for embryogenesis, a viable organism) remain the same despite internal and external perturbations [11–13]. A developmental endpoint can also be robust despite variability in intermediate processes or phenotypes, and variability of this kind constitutes plasticity (the opposite of robustness) when the perturbation is environmental [14]. During embryogenesis, for example, genetic information from parents is merged and translated into patterning and growth, which jointly establish the various cell types, tissues and organs that transform a fertilized egg into an independent juvenile or larva [10,15]. Spatio-temporal regulation and coordination of these events sees embryos progress from single cells to increasingly complex, organized body plans and errors at any point in progression can cause defects or death [16,17]. Accommodating different temperatures that accelerate or decelerate developmental progression therefore requires mechanisms that regulate the relative timing of developmental stages. How this occurs during embryogenesis in marine ectotherms is not yet understood.

Developmental stages are distinct developmental events, marking key morphological changes in structure, function and organization, that have long been used to measure developmental progression and compare it among individuals [16], species [17] or higher taxa [18] that develop asynchronously. The progression of these stages can in turn enable comparisons of development, and identify temporal changes in robustness, across different environmental conditions that inherently affect development time [13,19,20]. In particular, comparing the progression of developmental stages over time and across environments can reveal which stages act to coordinate robustness and buffer development against environmental perturbations. Such coordinating stages are known as developmental milestones [17].

**Figure 1.**
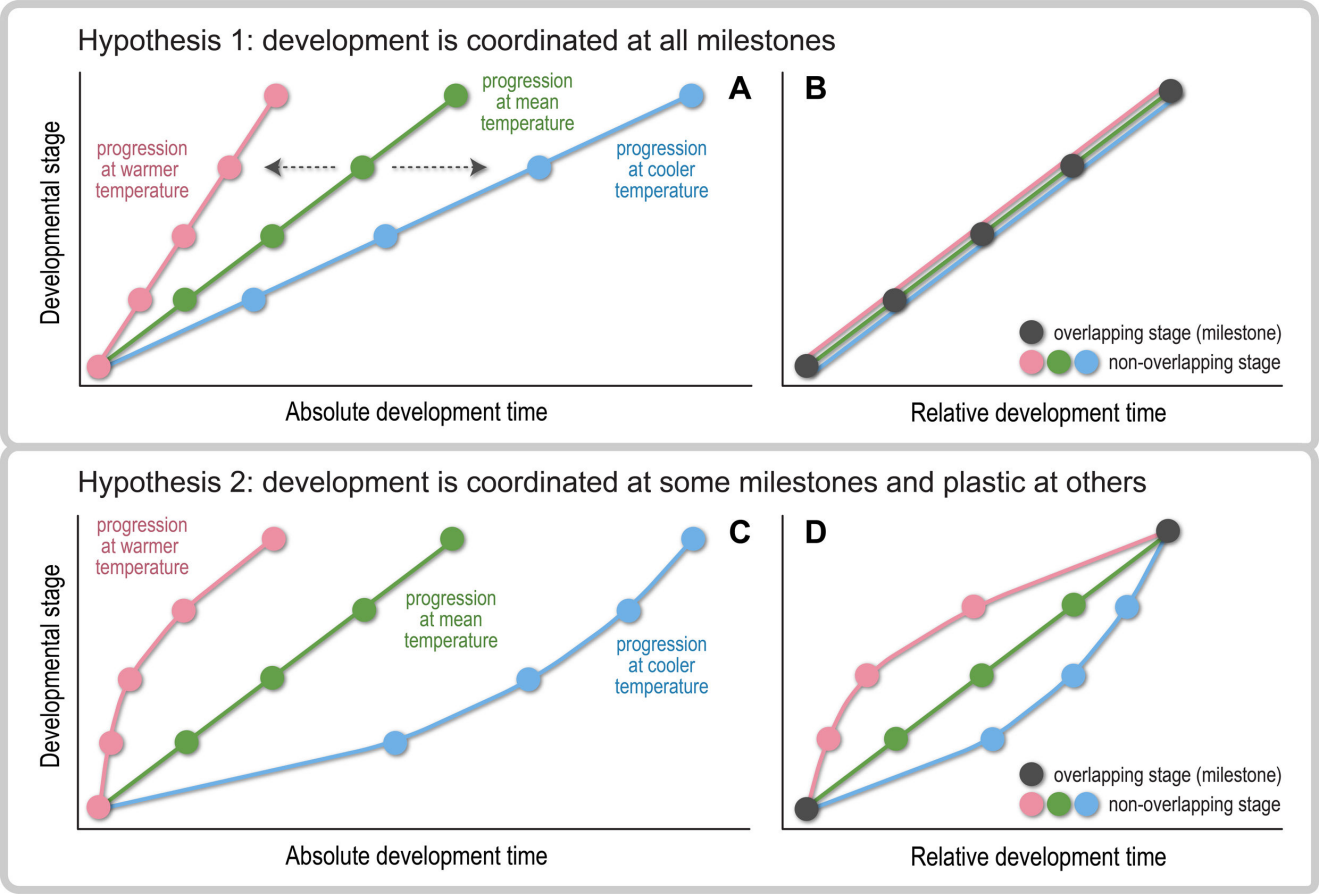
Alternative hypotheses to explain how developmental endpoints remain robust to environmental perturbations (e.g. changes in temperature) that affect development time. (A,B) In hypothesis 1, developmental progression in different environments is temporally coordinated at all developmental stages marking key changes in structure, function or organization. Consequently, (A) environmentally induced changes in absolute development time (A) accelerate (left arrow) or decelerate (right arrow) progression in a uniform manner, so (B) all stages overlap across environments when development times are normalized to the same relative scale. In this case, all stages are coordinated in relative time and represent developmental milestones. (C,D) In hypothesis 2, developmental progression is coordinated at particular milestones only. In this case, (C) changes in absolute development time alter progression in a nonuniform manner, and (D) only stages that overlap across environments in relative time are milestones*.* Between milestones, stages are plastic in their progression, so they do not overlap in relative time. Adapted from Oliveira *et al.* [13]*.*

Working on fruit fly larvae, Oliveira *et al.* [13] proposed two hypotheses to explain how developmental endpoints remain robust to environmental perturbations. First, robustness could be achieved by tightly coordinating developmental progression at all stages to the endpoint. Under this hypothesis, environmentally induced changes in absolute development time accelerate or decelerate the timing of developmental stages at the same rate (figure 1A), known as uniform or proportional scaling [20,21]. Progression should then be conserved across environments when assessed on the same, relative time scale (i.e. normalized to the endpoint by environment; figure 1B), and all stages act as developmental milestones since all stages are coordinated in relative time. Second, robustness could be achieved by coordinating developmental progression only at particular milestones, predicting that rates of progression at other stages should drift apart with environmentally induced changes in development time (figure 1C). In this case, the developmental endpoint is robust to environmental change, but developmental trajectories differ plastically across environments, in relative time (figure 1D).

The hypotheses above offer a valuable starting point for investigating how temperature affects the progression of milestones in embryogenesis. To our knowledge, this has rarely been done except in model systems such as fruit flies and nematodes (but see [16]). The timing of embryonic stages in diverse *Drosophila* fruit fly species, for example, scales uniformly across temperatures, at least until thermal limits for viability are met [20,22]. In contrast, *Drosophila melanogaster* wing patterning is coordinated at developmental milestones at the beginning and end of the final larval instar, but developmental progression is thermally plastic during the intervening stages [13]. In nematodes, however, developmental stages scale uniformly with temperature during larval development [23] and deviate from uniformity during embryogenesis [24]. These findings argue that as temperature accelerates or decelerates development, the coordination of key events can vary due to lineage-specific adaptation or constraints on developmental robustness at different life stages. Such insights are yet to extend to marine external fertilizers.

Here, we investigate the effects of temperature on embryogenesis in the marine tubeworm, *Galeolaria caespitosa* (named by genus hereafter). Like most marine ectotherms, *Galeolaria* spawns gametes of both sexes directly into the sea for external fertilization and development [25,26], which occur year-round along the coast of temperate Australia—some of the fastest warming waters globally [27,28]. To explore how embryos maintain robust development in the face of perturbation by temperature, we first develop a scheme for staging the progression of development based on confocal imaging of fluorescently labelled cortical F-actin and nuclear DNA. We then sample, label and stage embryos hourly throughout embryogenesis at the minimum temperature of the coldest month (11°C), annual mean temperature (17°C) and maximum temperature of the warmest month (22°C) in nature, to compare developmental progression across temperatures that dramatically alter development time. We find that progression is largely conserved across temperatures when normalized to development time, but earlier milestones are less robust to warming than later ones. Our results suggest that embryos achieve robustness to thermal perturbation by tightly coordinating the relative timing of developmental stages in embryogenesis, offering clues to how embryos of many marine species may respond to ongoing climate change.

## 2. Methods

### 2.1. Study species and sampling

*Galeolaria* (family Serpulidae) is a marine ecosystem engineer of rocky shores across temperate Australia, building reef-like masses of calcareous tubes that provide habitat and reduce abiotic stress for associated communities [29,30]. *Galeolaria* is a typical external fertilizer whose embryos and larvae disperse passively in currents and develop in direct contact with ambient sea temperatures [26,31]. The thermal range for survival narrows from fertilization (approx. 0.5°C–36°C) to embryogenesis (appxox. 10°C–28°C), but changes little with progression to larval development [9]. The thermal optimum for embryo survival is approximately 19°C and shifts in temperature at this stage shift the larval optimum (approx. 18°C–21°C) to match [9,32]. This thermal ecology points to embryos as thermal bottlenecks in early life and makes *Galeolaria* an excellent system for investigating how the developmental stages of embryogenesis—enigmatic in most species and external fertilizers especially—respond to temperature.

We sampled adult *Galeolaria* between August and October 2019 from a single population at Brighton, Victoria, Australia. Here, sea temperature falls to approximately 11°C in the coldest month of the year, rises to approximately 22°C in the warmest month and ranged from approximately 11°C to 16°C in the sampling period [33]. Adults were transferred to oxygenated seawater tanks at Monash University, held for 2 h at their natural temperature to limit transfer stress, then adjusted to approximately 17°C and acclimated for two weeks on an ad libitum diet of microalgae refreshed every other day. Seawater salinity was monitored to ensure it remained at natural levels.

### 2.2. Gamete collection and fertilizations

Fertilizations were done in blocks, using sperm pooled from eight males and eggs pooled from eight females to minimize male–female compatibility effects [34]. Each adult was removed from its tube and placed in 1 ml of fresh 0.22 µm filtered seawater to spawn, then its gametes were collected and pooled with others of the same sex. Pooled sperm were diluted to 10^7^ cells per ml (which maximized fertilization success in pilot work), while pooled eggs were kept at their ambient dilution (approx. 1500 cells per ml) since density of the limiting gamete does not affect fertility [35,36]. Fertilizations were initiated by combining 18 ml of sperm solution and 2 ml of egg solution for 20 min (which produced at least 80% fertilization success in pilot work), then ended by thoroughly rinsing vial contents through 55 µm mesh with filtered seawater to remove sperm and unfertilizable eggs (which are smaller than 55 µm in our study population; [37]). Fertilizations were maintained at approximately 17°C to ensure that our manipulation of temperature (see below) explicitly targeted embryogenesis, with all else being equal beforehand.

### 2.3. Manipulation of temperature during embryogenesis

We examined embryogenesis at three temperatures, 11°C, 17°C and 22°C, in an unbalanced block design with 12−48 vials of embryos per block and 9 blocks in total. The first 5 blocks had replicate vials at all temperatures, while remaining blocks had vials at 11°C only since embryos took substantially longer to develop at this temperature (75 h, compared with 32 h at 17°C and 28 h at 22°C). Temperatures were chosen to approximate the annual mean and extremes in nature, with the lower extreme being most deleterious to embryo survival (approx. 10% at 11°C compared with approx. 30% at 22°C and approx. 45% at 17°C [9]). Temperatures were also held constant, given evidence that daily fluctuations (the temporal scale relevant to embryogenesis) have little effect on biological responses of ectotherms compared with means and extremes [38].

Vials held 3 ml of filtered seawater, were loosely capped (though oxygen is not limited at this volume [36]) and were suspended upright in water baths maintained at designated temperatures (±0.1°C) by immersion heaters. Sampling of embryogenesis was initiated by pipetting 30 putative zygotes into each vial immediately after fertilization. Two vials per temperature were then sampled hourly until embryogenesis was complete, with sampled vials placed immediately on ice to stop development. Embryos were then fixed, fluorescently labelled for F-actin and nuclear DNA to reveal their internal structures and organization and imaged using confocal microscopy to stage developmental progression in each one (see §2.4 below). We staged 8−10 embryos per vial (though two vials had only 2−4 embryos) and discarded the rest, giving a total of 2404 staged embryos (1282 at 11°C, 604 at 17°C and 518 at 22°C) from 276 vials by the end of the experiment.

### 2.4. Fluorescent labelling and imaging of embryos

Embryos in each vial were permeabilized for 4 min in a 1:1 solution of 1 M sucrose and 0.25 M sodium citrate, then rinsed and fixed overnight at 4°C in a solution of 4% paraformaldehyde and 0.1% TritonX-100 in phosphate-buffered saline (PBS). After fixation, embryos were rinsed and washed 4 times in PBT (0.3% TritonX-100 in PBS) for 10−15 min per wash, then incubated consecutively with fluorescent probes labelling F-actin and DNA. For F-actin labelling, embryos were incubated for 20 min in a 1:15 solution of ActinGreen 488 ReadyProbes Reagent (Invitrogen, R37110) in PBT, then rinsed and washed twice in PBT. For DNA labelling, embryos were incubated for 5 min in a 1 : 50 solution of DAPI 4',6-diamidino−2-phenylindole (Invitrogen, D1306) in distilled water, then rinsed and washed 3 more times in PBT. To prevent loss of fluorescence, embryos were stored in PBT in the dark for no more than 4 days before imaging.

**Figure 2.**
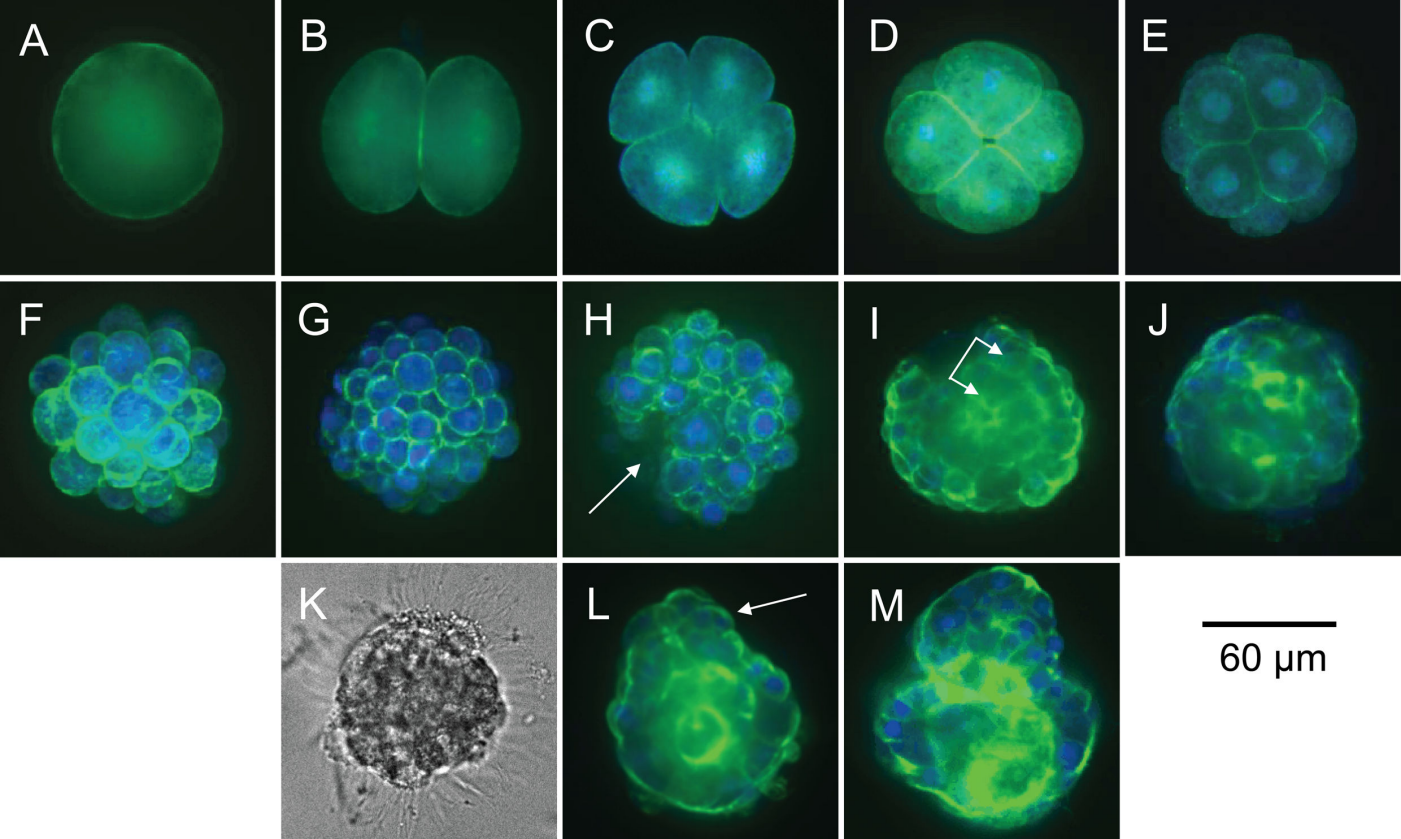
Developmental stages in embryogenesis, identified by fluorescent labelling of F-actin (green) and nuclear DNA (blue) or phase-contrast optics (grey). (A) Fertilized egg or zygote. (B) 2-cell embryo (first division). (C) 4-cell embryo (second division). (D) 8-cell embryo (third division). (E) 16-cell embryo (early morula). (F) 32-cell embryo (late morula). (G) 64-cell embryo (blastula) with incipient fluid-filled cavity (blastocoel) signalling onset of body patterning. (H) Invagination (at arrow) signalling onset of morphogenesis (gastrulation and tissue formation). (I) Early gastrula with emergent tissues in distinct germ layers (at arrows). (J) Late gastrula with complete gut. (K) Appearance of equatorial cilia (prototroch) used in locomotion. (L) Formation of apical sensory organ (at arrow). (M) Free-swimming trochophore larva ready to feed.

Embryos were imaged using an Olympus CV1000 spinning disc confocal microscope, capturing optical sections at 1 μm intervals with excitation wavelengths of 488 nm for ActinGreen 488 and 405 nm for DAPI. Images were processed and analysed using ImageJ [39] to form a detailed, three-dimensional image of each embryo and its internal structures. Since embryos also develop external bands of locomotory cilia (prototrochs) that do not fluoresce, these structures were imaged using phase-contrast optics. Embryos were then categorized into developmental stages based on the structures expressed when embryos were sampled at different temperatures (figure 2). Our choice of sampling times and labels meant that stages included structural changes from first division to completion of the larval body plan but excluded pre-division processes (e.g. activation of metabolism) in the minutes after fertilization.

## 3. Statistical analyses

Data were analysed in linear mixed-effects models, fitted using Bayesian Markov chain Monte Carlo (MCMC) estimation in the *MCMCglmm* package [40] for R 4.4.0 [41]. We used Bayesian estimation because data were very unbalanced among blocks (see §2.3), stage was an ordered categorical response that violated assumptions of normality and equivalent likelihood-based models failed to converge. All aspects of model fitting followed standard recommendations [40,42]. Fixed effects had default weak priors (normal posterior distributions with means of 0 and variances of 10^8^ on the link scale). Random effects had scaled *F*_1,1_ priors set by parameter expansion, and residual variances had flat priors or were fixed at 1 for ordinal models (see below). Alternative priors were explored and affected the speed of model convergence (identified by stationary traces with autocorrelations of <0.1 between all samples) but not final results. Posterior distributions for all parameters were estimated from 6 100 000 MCMC iterations sampled every 3000 iterations after a burn-in of 100 000 iterations, giving 2000 posterior samples per parameter.

### 3.1. Effects of temperature on developmental progression

We analysed the progression of stages at each temperature in ordinal mixed-effects regression models with cumulative probit link functions (known also as threshold models [42]). Such models account for the mean–variance relationship typical of categorical responses by assuming normality on the link scale [42] and are appropriate when categories are ordered, but their spacing is unknown [43], as is the case for developmental stages. Developmental stages were modelled as a cubic function of development time (hours post-fertilization) based on preliminary curves fitted to raw data using unconstrained (locally estimated scatterplot) smoothers. Trends from ordinal regressions are interpreted no differently to those from standard regressions, and estimate changes in developmental stage (on the link scale) per unit change in development time, holding other trends constant. Since development time was mean-centred by temperature to improve model performance and interpretability [44], linear trends estimate the average slopes relating developmental stage to time, while quadratic and cubic trends estimate the degree to which slopes are steeper or shallower at extremes of time [44]. Temperature and its interactions with trends were modelled as additional fixed effects, while block and vial within blocks were modelled as random effects.

First, we fitted a model with development time on the absolute scale (i.e. mean-centred by temperature, but still in units of hours). Second, we fitted a model with development time on the relative scale (i.e. mean-centred by temperature and normalized by the maximum hours to the endpoint per temperature, converting units to percentages of development) to explore progression accounting for differences in absolute time. The shapes of the resulting curves in relative time allowed us to determine if all developmental stages were milestones (indicated by curves of similar shape) or only some stages acted as milestones (causing shapes to diverge across temperatures). Finally, we tested the effects of time, temperature and time × temperature interaction using omnibus Wald *χ*^2^ tests (computed in the *VCVglmm* package [45]) and interpreted trends at each temperature as significant if their 95% credible intervals (computed in the *emmeans* package [46]) excluded zero. Groups within significant effects were contrasted pairwise in *emmeans* and interpreted as significantly different if contrasts had 95% credible intervals that excluded zero.

### 3.2. Effects of temperature on variability during developmental progression

**Figure 3.**
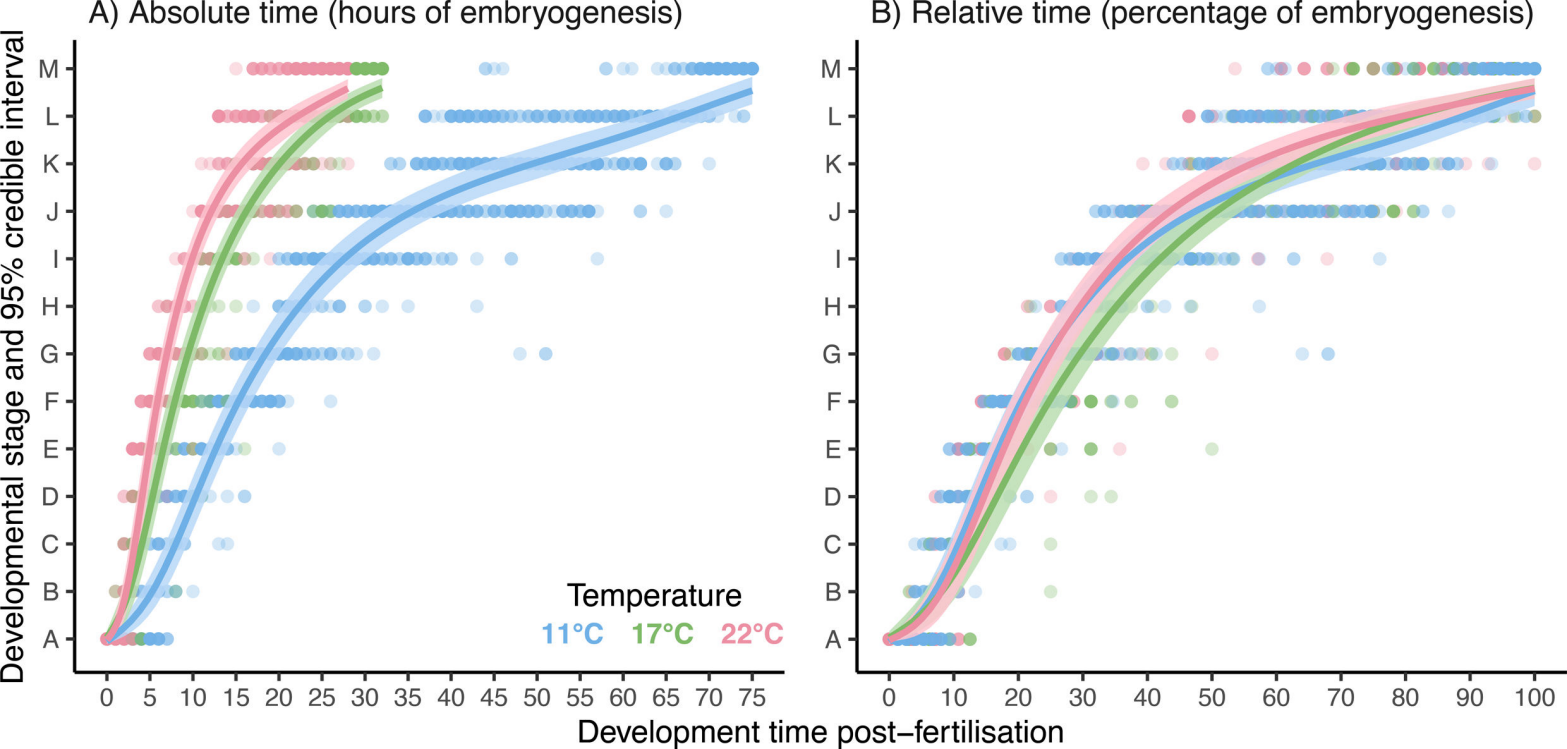
Effects of temperature on the progression of developmental stages in embryogenesis, with development time (A) on the absolute scale (hours of embryogenesis) and (B) on the relative scale (percentage of embryogenesis). Curves are posterior means predicted by ordinal mixed-effects regressions and shaded areas are 95% credible intervals around means. Points are 2404 individually staged embryos and their time of sampling (raw data), with 1282 embryos sampled hourly for 75 h at 11°C, 604 embryos sampled hourly for 32 h at 17°C and 518 embryos sampled hourly for 28 h at 22°C. Embryos were sampled in 276 replicate vials (2 vials per hour per temperature) spread across 9 blocks (5 blocks had vials at all temperatures and remaining blocks had vials at 11°C only; see details in methods).

We analysed temporal variability in developmental progression at each temperature in linear mixed-effects models with temperature, milestone and their interaction as fixed effects and block as a random effect. Variability was analysed on both absolute and mean-standardized scales (see below), after raw data (points in figure 3A) showed that it increased with development time. Mean-standardization solves this problem [47] and allowed us to compare variability among groups sampled at different times (milestones per temperature and vice versa), all else being equal. Replicate measures of variability on each scale were computed by summarizing the raw times at which each milestone was sampled in each temperature-block combination. For each combination, we computed the absolute variability per milestone as the standard deviation of times when it was sampled, then divided by the mean time to compute the mean-standardized variability (coefficient of variation) per milestone. This gave 103 measures on each scale (44 at 11°C, 31 at 17°C and 28 at 22°C) for analysis. As above, we tested the effects of milestone, temperature and milestone × temperature interaction using omnibus Wald *χ*^2^ tests (computed in *VCVglmm*). Groups within significant effects were contrasted pairwise (for temperatures) or consecutively (for milestones, which progress directionally) in *emmeans* and were interpreted as significantly different if contrasts had 95% credible intervals that excluded zero.

## 4. Results

Based on structures identified by fluorescent labelling and imaging, embryos were categorized into 13 developmental stages (A–M in figure 2) spanning embryogenesis at each temperature. These were zygote formation (figure 2A), the first five cell divisions of the early embryo including early and late morulae (figure 2B*–*F), the blastocyst stage (figure 2G), early through late gastrulation (figure 2H*–*J), the appearance of locomotory cilia (figure 2K), formation of an apical sensory (figure 2L) and the endpoint of a free-swimming larva ready to feed (figure 2M). Stages were chosen to sample representative points in the ordered sequence of cell division, patterning and morphogenesis that characterizes animal embryogenesis, using descriptions for *Galeolaria* and other serpulid tubeworms [48–50] to stage lineage-specific features such as gain of independent locomotion and feeding.

### 4.1. Effects of temperature on developmental progression

At all three temperatures, embryos progressed faster though early developmental stages and slower through later ones, producing sigmoid developmental trajectories (figure 3). On both time scales analysed, trajectories were defined by positive linear and cubic trends and negative quadratic trends that interacted with temperature (table 1) and differed significantly from zero (except for cubic trends at 17°C; electronic supplementary material, figure S1). Estimates of random effects were negligible (electronic supplementary material, figure S2).

**Table 1.** Effects of temperature on the progression of developmental stages in embryogenesis, related to development time by linear, quadratic and cubic trends. Wald *χ*^2^ statistics are tests of fixed effects estimated by ordinal mixed-effects models, with development time on (A) the absolute scale (hours of embryogenesis) and (B) the relative scale (percentage of embryogenesis). Random effects are presented in the electronic supplementary material, figure S2.

fixed effects	Wald χ^2^	d.f.	*p*
*(A*) *absolute development time (hours of embryogenesis*)			
linear trend for time	280.11	1	<0.01
quadratic trend for time	150.92	1	<0.01
cubic trend for time	15.09	1	<0.01
temperature	24.22	2	<0.01
temperature × linear trend for time	85.07	2	<0.01
temperature × quadratic trend for time	103.73	2	<0.01
temperature × cubic trend for time	11.61	2	<0.01
*(B) relative development time (percentage of embryogenesis*)			
linear trend for time	333.26	1	<0.01
quadratic trend for time	157.49	1	<0.01
cubic trend for time	32.96	1	<0.01
temperature	23.87	2	<0.01
temperature × linear trend for time	8.50	2	0.01
temperature × quadratic trend for time	12.83	2	<0.01
temperature × cubic trend for time	7.44	2	0.02

**Figure 4.**
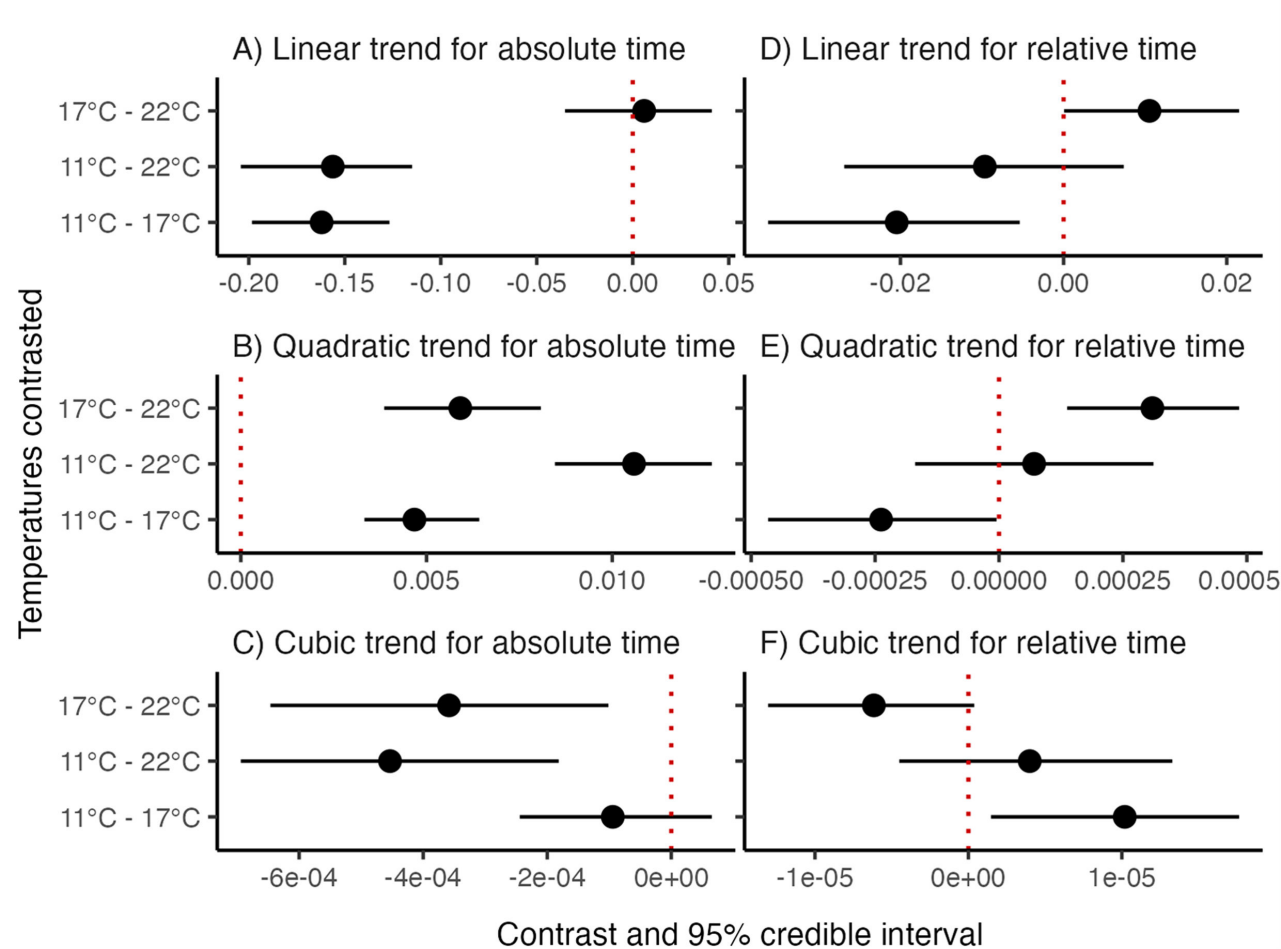
Linear, quadratic and cubic trends in rates of developmental progression contrasted between temperatures. Contrasts subtract trends at warmer temperatures from trends at cooler ones, so negative estimates mean that warming strengthens trends and positive estimates mean the opposite. Panels on the left (A–C) contrast trends for absolute development time (hours of embryogenesis) and panels on the right (D–F) contrast trends for relative development time (percentage of embryogenesis). Trends differ significantly between temperatures if their contrasts have 95% credible intervals that exclude zero, marked by red dotted lines.

On the absolute time scale (hours of embryogenesis; figure 3A), embryos took markedly longer to complete development at 11°C (75 h maximum) than at 17°C (32 h maximum) and 22°C (28 h maximum). Accordingly, the average rate of progression estimated by linear trends was similar at 17°C and 22°C but significantly lower at 11°C (figure 4A). Successive gains in temperature did, however, make quadratic trends more negative (figure 4B) and cubic trends more positive (but not significantly so from 11°C to 17 °C; figure 4C). Hence, warming accelerated progression faster at early stages and decelerated it faster at later ones to produce the similar rates of progression, on average, at 17°C and 22°C.

On the relative time scale (percentage of embryogenesis; figure 3B), developmental trajectories showed only modest thermal sensitivity, no longer differing between 11°C and 22°C (figure 4D–F) and differing only subtly between these temperatures and 17°C. Specifically, progression accelerated and decelerated faster—that is, quadratic trends grew more negative (figure 4E) and cubic trends more positive (but only marginally so from 17°C to 22 °C; figure 4F)—at more extreme temperatures. Overall, however, the substantial overlap of trajectories across temperatures was most in line with our first hypothesis (figure 1B). We therefore refer to developmental stages as milestones from this point forward.

### 4.2. Effects of temperature on variability during developmental progression

**Figure 5.**
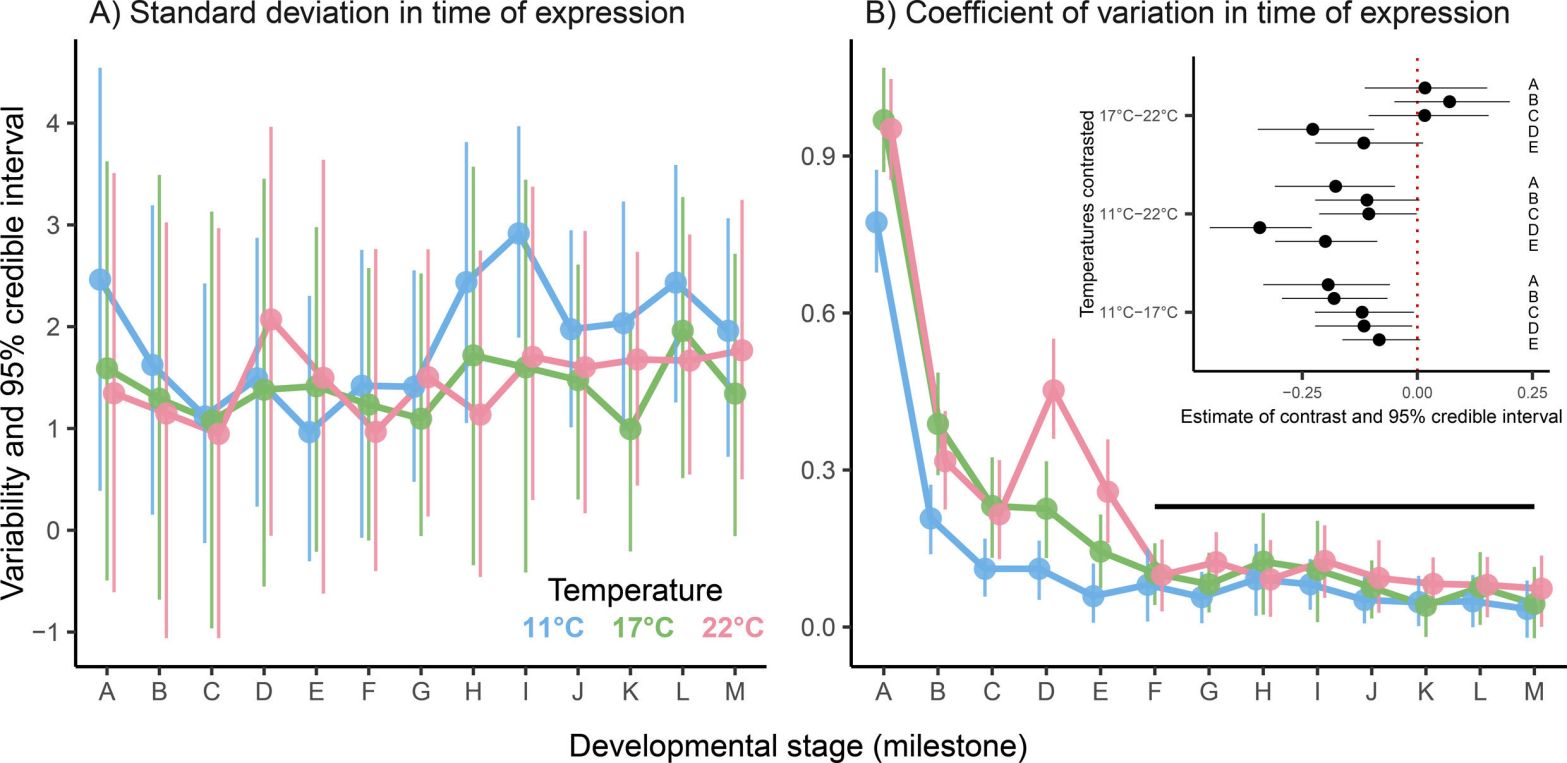
Effects of temperature on variability during developmental progression, (A) on the absolute scale (standard deviation in the time of milestone expression) and (B) on the mean-standardized scale (coefficient of variation in the time of milestone expression). For each scale, 103 measures (44 at 11°C, 31 at 17°C and 28 at 22°C) were obtained for analysis by summarizing the raw times at which developmental stages representing milestones were sampled (horizontal values of points in figure 3A). In (A), no effects are significant. In (B), variability differs significantly between early milestones (A–E) but not later ones (F–M, under the black line). Also in (B), variability differs significantly between temperatures at early milestones whose contrasts and 95% credible intervals exclude zero (marked by the red dotted line in the inset) but not at later milestones (omitted from the inset). Contrasts in the inset subtract variability at warmer temperatures from variability at cooler ones, so negative estimates mean that warming increases variability (or decreases robustness) during development.

Temporal variability during developmental progression depended on the temperature, milestone and time scale considered (random effects were again negligible; electronic supplementary material, figure S3). Variability on the absolute scale (standard deviation in time of expression) was unaffected by temperature or milestone (figure 5A; table 2A). We note, however, that contrasts of groups in each factor sampled at different times were confounded by the increase in variability over time (see the spread of raw data in figure 3A).

**Table 2.** Effects of temperature and milestone on variability during developmental progression. Wald *χ*^2^ statistics are tests of fixed effects estimated by linear mixed-effects models, with variability (A) on the absolute scale (standard deviation in the time of milestone expression) and (B) on the mean-standardized scale (coefficient of variation in the time of milestone expression). Random effects are presented in the electronic supplementary material, figure S3*.*

fixed effects	Wald *χ*^2^	d.f.	*p*
*(A*) *standard deviation in time of expression*			
temperature	2.20	2	0.33
milestone	5.67	12	0.93
temperature×milestone	6.50	24	0.99
*(B) coefficient of variation in time of expression*			
temperature	46.41	2	<0.01
milestone	738.49	12	<0.01
temperature × milestone	45.05	24	<0.01

Variability on the mean-standardized scale (coefficient of variation in time of expression, correcting for the association with development time) was sensitive to both factors combined (figure 5B; table 2B). Based on contrasts of groups in each factor, this sensitivity was confined to early embryogenesis (milestones A–E, spanning zygote formation to the early morula stage; figure 5B) and was more prolonged and extreme at 22°C than other temperatures. Specifically, variability in the timing of early milestones fell sharply from zygote formation (milestone A) to the first cell division (milestone B), then more gradually from the first division to the second division (milestone C). At 11°C and 17°C, variability stayed low thereafter (contrasts among milestones C–M were non-significant). At 22°C, however, it spiked again at the eight cell stage (milestone D) before subsiding by the late morula stage (contrasts among milestones F–M were non-significant). In the window beforehand, milestone expression was less variable (more robust) at 11°C than other temperatures but differed little between 17°C and 22°C except for the spike at milestone D (figure 5B, inset). Overall, this suggests that embryogenesis may be perturbed by temperature initially but becomes increasingly robust and regulated as it progresses.

## 5. Discussion

In a fast warming world, embryos must be robust to changes in temperature causing profound changes in development time. This is especially vital for marine ectotherms that develop in direct contact with the external environment, yet how their embryos achieve robustness against thermal perturbation is unknown. Here in the marine tubeworm *Galeolaria*, we developed a scheme to stage embryos, then compared the progression of developmental stages across the annual temperature range (11–22°C) in nature. Despite a nearly threefold decrease in development time across temperatures, we detected only subtle changes in progression once stages were normalized to development time. This infers that all stages are developmental milestones acting to coordinate robustness, and that greater changes in the relative timing of milestones are non-viable. Deeper analysis of variability (plasticity) during progression showed that robustness (i) increases as embryos progress beyond early cell divisions and enter the morula, blastocyst and morphogenetic stages, and (ii) is perturbed by warming prior to the morula stage. Collectively, our results argue that embryos remain robust to changing temperatures by tightly coordinating events throughout development, but unknown mechanisms (possibly genetic, biophysical or endocrine in origin) enforce tighter coordination at later milestones especially.

It is unclear why development is sometimes tightly regulated throughout its progression and at other times regulated only at specific milestones. Similar to *Galeolaria* embryos, embryogenesis in *Drosophila* species scales uniformly with temperature, suggesting that development is also tightly regulated throughout [20,22]. Larval development in nematodes, measured by stem cell divisions in the hypodermis, shedding of the larval cuticle, and oscillation of *wrt* expression, also follows the mode of tight temporal regulation [23]. This contrasts with the temporal scaling of wing patterning during larval development across the viable temperature range in *D. melanogaster* [13], where development is coordinated only at two specific milestones in the final larval stage. The difference in outcomes could simply reflect the milestones chosen for study in each system, including *Galeolaria*. Alternatively, tight regulation of patterning in time and space might be the norm for embryos, whereas various paths can lead to a robust final pattern for larvae [10]. That the progression of embryogenesis in *Galeolaria* was largely, but not completely, conserved across temperatures in relative time, suggests that the limits of regulation may be stretched by current thermal extremes. More work is needed to understand if those limits risk being exceeded with ongoing climate change.

A closer look at temporal variability during developmental progression at different temperatures implies that *Galeolaria* embryos are more robust later in embryogenesis than earlier on. All else being equal (i.e. scaled by the mean time of milestone expression per temperature), expression grew markedly more synchronous as embryos progressed from early cell divisions to the morula stage (figure 5B). Temporal variability during early divisions could in part result from our sampling protocol, which examined milestones every hour, potentially under-sampling early stages and increasing variability at those examined. However, this does not explain all patterns that we observe. The first description of early embryogenesis in *G. caespitosa* reports that the first cell division (milestone B) occurs approximately 90 min after fertilization in embryos reared at 25°C [49]. At that temperature, each of the second to fifth divisions (milestones C–F) occur approximately 30 min apart, and embryos reach the blastocyst stage (milestone G) 4−5 h after fertilization [49]. Our data show that development slows at lower temperatures, so we expect early cell divisions to take longer at the temperatures used in our study. More importantly, since milestones C–F occur at even time intervals, under-sampling cannot explain the heat-induced spike in variability at the eight cell stage (milestone D).

It could also be argued that later milestones involving blastocyst and tissue and organ formation are inherently more robust than early cell divisions, due to the greater control of growth and pattern needed for proper development of increasingly complex structures [15]. It could further be argued that, in *Galeolaria*, the timing of embryonic milestones is controlled by a shared mechanism tied to development time and activated after cell divisions. Kuntz & Eisen [20] reported something similar in fruit flies, attributing it to unknown molecules whose action or abundance changes in a clock-like way to schedule the events that turn fertilized eggs into larvae or (more likely) a shared rate-limiting process such as energy use or production. If the latter mechanism applies here, then the onset of temporal robustness in embryos across temperatures could coincide with a shift in energetics, from being fuelled primarily by maternal energy stores (yolk) to being fuelled by energy production in embryos themselves [51].

Energetic costs of development could also explain why the progression of embryogenesis in *Galeolaria* was more perturbed by warming at early milestones than later ones. In theory, such costs decrease with shorter development time, lower metabolic rate or both [52,53]. That warming visibly reduced temporal robustness at early milestones, independent of reducing development time (figure 5B), may therefore point to metabolic instability during early cell division. Metabolic rate rises with temperature before falling steeply as upper thermal limits are met [54,55]. It also increases rapidly as embryos start dividing, stabilizes as divisions give way to cellular rearrangements at gastrulation, then varies according to species’ development modes [51]. Together with our results, this would imply that the energetics of early division set the upper thermal limits of embryogenesis in ectotherms—an idea supported by the temperature dependence of the first embryonic division in nematodes [24]. More data are needed to evaluate the idea more broadly: to our knowledge, only one other study has measured the temporal robustness of embryogenesis at different temperatures (Chong *et al.* working on fruit flies [22]), but did not consider milestones before gastrulation.

In summary, embryogenesis is often viewed as one of the most fragile stages of life yet still produces proper cell patterning, setting up the same body plans and functional structures vital for survival and reproduction, across diverse real-world environments [8,10]. Using an exemplar marine ectotherm with external fertilization and development, we have shown that embryogenesis ensures properly patterned progeny across a broad temperature range by largely conserving developmental progression apart from temperature-induced changes in development time. Further, we have shown that embryogenesis is most prone to perturbation by temperature in the window before gastrulation but becomes increasingly robust to temperature as development progresses. Precisely how this impacts survival during the course of embryogenesis, and how embryos regulate their development to ensure robust patterning in the face of changing temperatures, remain unknown. Unravelling the underlying mechanisms driving this robustness thus remains a critical frontier, with implications extending from fundamental principles of developmental biology to species’ responses to climate change in marine systems.

## Data Availability

Data and R code supporting this article have been made available as electronic supplementary material [56].
